# Improvements in Modern Surgery

**Published:** 1852-08

**Authors:** 


*Improvements in Modern Surgery.*—Dr. Mussey in his last *Introductory Lecture*, enumerates some of the chief improvements made in surgery during the present century. He says:

In the healing of *simple wounds*, water dressings may often be so managed as to effect an entire union of the divided parts without suppuration, and this, too, where the divided parts are not kept in contact, as is necessary for the adhesive process, the space being filled up by the requisite vital materials without suppuration. This has been called the modeling process, by Dr. Macartney.

Certain poisoned wounds: the bite of the rattlesnake, it has been alleged on pretty good authority, has been cured by the early local application of tincture of iodine.

Tetanus (traumatic): a number of well authenticated cases have been successfully treated by chloroform.

Erysipelas: locally, iodine, nitrate of silver; internally, carbonate of ammonia, opium, and quinine. In phlegmoid erysipelas, as well as carbuncle, incisions are of great value.

Anæsthtic agent: a priceless boon, recently handed down to us by a kind Providence to disarm surgery of its terrors. In my practice ether and chloroform have been used without injury, in over six hundred operations. In St. Bartholomew's Hospital, in London, according to Mr. Skey, in a statement made last summer, chloroform had been employed, without injury, in between nine thousand and ten thousand operations. The French Academy awarded for this discovery, to our countrymen, Doctors Jackson and Morton, each a medal; the one for having made the suggestion, the other for having put the suggestion to experiment.

In surgical anotomy, great process has been made. This is exemplified in the anatomy of the neck, in reference to the extraction of tumors and the ligating of vessels. The anatomy of hernia has arrived at wonderful precision, compared with what was known a century ago. Various accompaniments of strangulated hernia—difficulties to be encountered, and means of meeting them—are far more clearly understood.

Surgery of the Arteries. This department has undergone such important modern improvements, as almost to admit of its origin being dated about the time of Hunter. Ligating an artery on the cardiac side of aneurismal tumor has been chiefly practiced. The artery, too, has been successfully ligated on the distal side of the tumor, as recommended by Brasdor. Success, too, has repeatedly followed the compression of the artery on the cardiac side of the tumor; and even on the distal side, as reported by Dr. Goldsmith, of Vermont. In a recent aneurism, according to Dr. Dudley, a compress upon the tumor, supported by a bandage upon the whole limb, has effected a cure.

In 1845, the subclavian artery had been litigated *sixty-nine times:* of this number there were thirty-six recoveries and thirty-three deaths.

Dupuytren and Liston, each has tied successfully the subclavian artery, under cover of the anterior scalenus muscle.

The aorta has been ligated *four* times, the arteria innominata *nine* times, and the subclavian, on the tracheal side of the scaleni muscles, *five* times. Death followed in every case, leaving surgeons little room to hope for any better result from a repetition of either of these operations.

The surgery of the eye has been greatly improved by Scarpa, Maunoir, Adams, Lawrence, Mackenzie, Tyrrel, Alexander, Roux, Velpeau, Sichel, Desmarses, Cuvier, Rogers, Delafield, Pancoast, Hays, and a host of others.

In the surgery of the ear, Itard, Deleau and Kramer, have been distinguished.

Plastic surgery has undergone important modern improvements. Taliacotius, of Bologna, in Italy, in the latter part of the sixteenth century, gained a temporary celebrity by his rhinoplastic operations, in which he formed a new nose by a flap of skin taken from the arm. This operation fell into disuse for a long period. Rhinoplasty, however, was revived and improved by Messrs. Carpue and Linn, of London. More recently, plastic surgery attracted the attention of numerous surgeons, as Græfe, Diefenbach, Frick, Zeis, Chelius, Delpech, Dupuytren, Lisfane, Jobert, Liston, J. M. Warren, Pancoast, Mutter, Post and Hullhen.

Staphyloraphy or palate suture was practiced with success by Roux, Græfe, Diefenbach, Warren, and others. This operation has been recently improved by Mr. Ferguson, of London, by dividing the levator palati and palato pharyngeal muscles.

In harelip operations, important improvement has been made, especially in those revolting cases with a double fissure and peninsular portion of the upper jaw connected with the septum of the nostrils, and projecting horizontally forward.

Tenotomy and Myotomy. Great progress has been made in the relief of deformities, as of club-foot, wry neck, strabismus, permanently contracted fingers.

In chronic enlargement of the tonsils, excision is now employed instead of ligation, which was used forty years ago.

Cancer of the tongue: nearly half of that organ has been successfully removed.

Excision of the upper and lower jaw: the latter operation has been performed by disarticulating the bone without dividing the facial nerve, or the duct of Steno, thus preserving the symmetry of the face.

In chronic abscess of bone, limbs are now saved by perforating the wall of the bone, which were formerly doomed to amputation.

In fractures of the limbs, the wet bandage of Dudley, in addition to splints, should not be overlooked, as marking improvement in the treatment.

In compound fractures, the collodion is of great value in excluding the air from the wound. In un-united fracture, or false joint, the seton, sawing the ends of the bones, subcutaneous scarification, lateral or longitudinal compression of the fragments, ivory pegs and wire bridles, and more especially a suitable constitutional treatment, have, in their turn, promoted cure.

In dislocations, anæthetic agents will probably take the place of copious bleedings, nauseating doses and the extreme warm bath.

In amputations the flap operation, as well as amputations at the hip, shoulder and ankle joints, show a progress in this department of surgery.

Diseases of the joint are better understood than formerly, and the treatment is more successful. Excision of the diseased elbow joint has been repeatedly successful.

Iodine injections in hydrarthus, in enlarged bursæ mucosæ, in ganglions, in spina bifida and chronic hydrocephalus, have been practiced with important benefit.

Injuries and diseases of the brain and spinal cord are much better understood.

